# The Dying Art of Vaginal Hysterectomy: A Novel Simulation

**DOI:** 10.7759/cureus.6362

**Published:** 2019-12-12

**Authors:** Jamie C Humes, Larissa Weir, Erin A Keyser, Maria M Molina

**Affiliations:** 1 Obstetrics and Gynecology, Brooke Army Medical Center, Fort Sam Houston, USA

**Keywords:** vaginal hysterectomy, hysterectomy simulation, difficult hysterectomy, hysterectomy training, vaginal morcellation, uterine morcellation, pelvis model, uterus model, sponge uterus, hysterectomy

## Abstract

The rate of vaginal hysterectomy has decreased despite the procedure being the preferred hysterectomy method according to the American College of Obstetricians and Gynecologists (ACOG). Physicians have reported that some of the main barriers to performing minimally invasive hysterectomy are the size and shape of the uterus, difficulty of accessibility to the uterus, and surgeons' lack of training and experience. A simulation model for vaginal uterine morcellation was created in an effort to increase surgeons' confidence and to encourage them to select vaginal hysterectomy for their patients.

The Conner model, where polyvinyl chloride (PVC) piping is used to simulate the pelvis and vaginal canal, was used as the basis for the pelvis. A medium-density fiberboard (MDF) was used as a base, while a PVC piping structure was used to stimulate the pelvis. The uterus was created from a peanut (car-wash) sponge that was carved into a triangle shape. The reusable MDF/PVC model was built in approximately one hour and cost under USD 30. The sponge uterus was built in approximately 10 minutes and cost under USD 2.

Senior residents and faculty who have previously performed uterine morcellation participated in our simulation. Resident physicians reported that they felt more confident in their skills after the simulation. Both resident and staff physicians reported that the model had created a realistic experience.

We created a novel model for vaginal uterine morcellation that was reported to be realistic in the initial investigation and increased confidence in the procedure for physicians. The model is easy to create, affordable, and partially reusable.

## Introduction

Hysterectomy is the second-most common surgical procedure in women in the United States. Forty-five percent of women in the US who are older than 70 years have had a hysterectomy [1]. According to the American College of Obstetricians and Gynecologists (ACOG), the vaginal approach is the preferred method for hysterectomy as it is associated with better outcomes. Unfortunately, this approach has decreased in popularity in recent years, with its frequency declining from 25% of all hysterectomies in 1998 to 17% in 2010 [2].

While there is no difference in rates of complications when compared with abdominal hysterectomy, the vaginal approach is associated with a shorter hospital stay, faster return to normal activity, better functional capacity, and improved pain assessment. Moreover, when compared with the laparoscopic approach, the vaginal approach has a shorter operating time, lower overall costs, and increased patient satisfaction [2].

There is a discrepancy between the recommendations from ACOG and the rates of vaginal hysterectomy. Einarsson et al. have reported that when it comes to themselves or their spouse, 55.5% of the providers chose vaginal hysterectomy as the preferred route, 40.6% chose laparoscopic, and 8% chose abdominal hysterectomy. This is despite the fact that 83.9% of hysterectomies performed by the respondents were abdominal hysterectomies [1]. Physicians have reported that the main barriers to performing minimally invasive hysterectomy are technical difficulties and complications. The study also stated that most gynecologists indicated interest in incorporating more minimally invasive techniques into their practice [1].

Some of the most common indications for hysterectomy are conditions that have a tendency for a larger uterus or adhesions, such as leiomyoma (51.4%), abnormal uterine bleeding (41.7%), and endometriosis (30%) [2]. These conditions may render vaginal hysterectomy more technically challenging in many patients. As one of the main cited barriers to performing vaginal hysterectomy is technical difficulties [1], it is proposed that implementing a simulation for challenging vaginal hysterectomies could increase physicians' confidence and encourage them to select vaginal hysterectomy for their patients.

## Technical report

**Figure 1:**
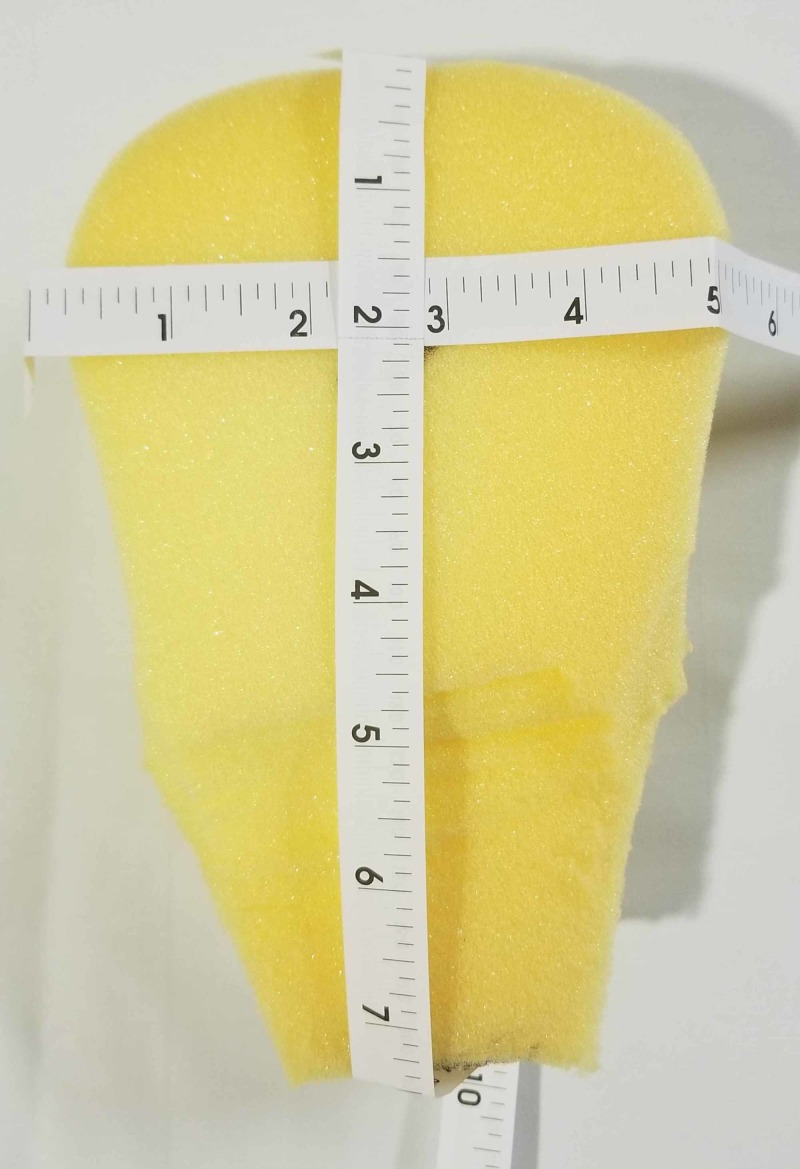
Dimensions of the sponge uterus

**Figure 2:**
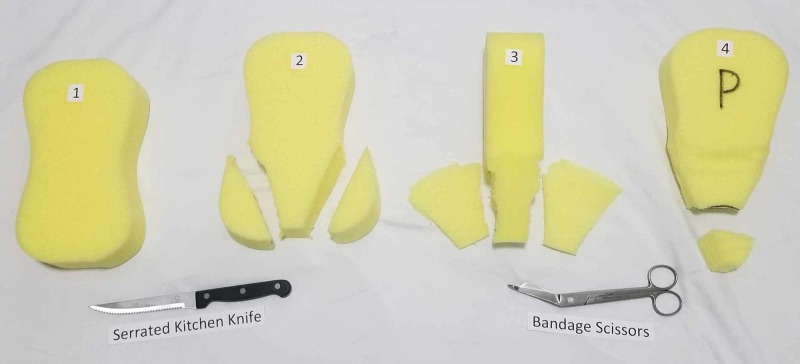
Creating the sponge uterus 1: sponge foam before modification; 2: two of the bulbous ends of the foam cut off diagonally using a serrated kitchen knife; 3: anterior and posterior surfaces cut in a tapered, wedge-like fashion; 4: anterior and posterior surfaces marked with permanent marker for identification

Our criteria in our selection of material to create the uterus for the purpose of simulation included affordability, ease of production, and reproducibility. Multiple modalities were experimented with to include formed foam, spray foam, ballistics gel, insulations, and silicone. While silicone was considered the most realistic in initial trials, it required the creation of a mold and was significantly more time-consuming and expensive at approximately USD 15 a uterus. For our model, the uterus was ultimately created from a formed foam, specifically a peanut (car- wash) sponge, which was carved into an approximately 4.5 x 4.5 x 5 cm of triangle shape (Figure 1). To carve out the foam, two of the bulbous ends of the foam were cut off diagonally using a serrated kitchen knife (Figure 2). The anterior and posterior surfaces were then cut in a tapered, wedge-like fashion (Figure 2). The remaining stump was truncated to provide a more realistic length. The carved edges were trimmed for cosmesis of the triangle with bandage scissors. On the tapered end, a cervix was drawn with a permanent marker. The anterior and posterior surfaces were then marked with a permanent marker with an A and a P for identification (Figure 2). The sponge uterus was built in approximately 10 minutes and cost under USD 2.

**Figure 3:**
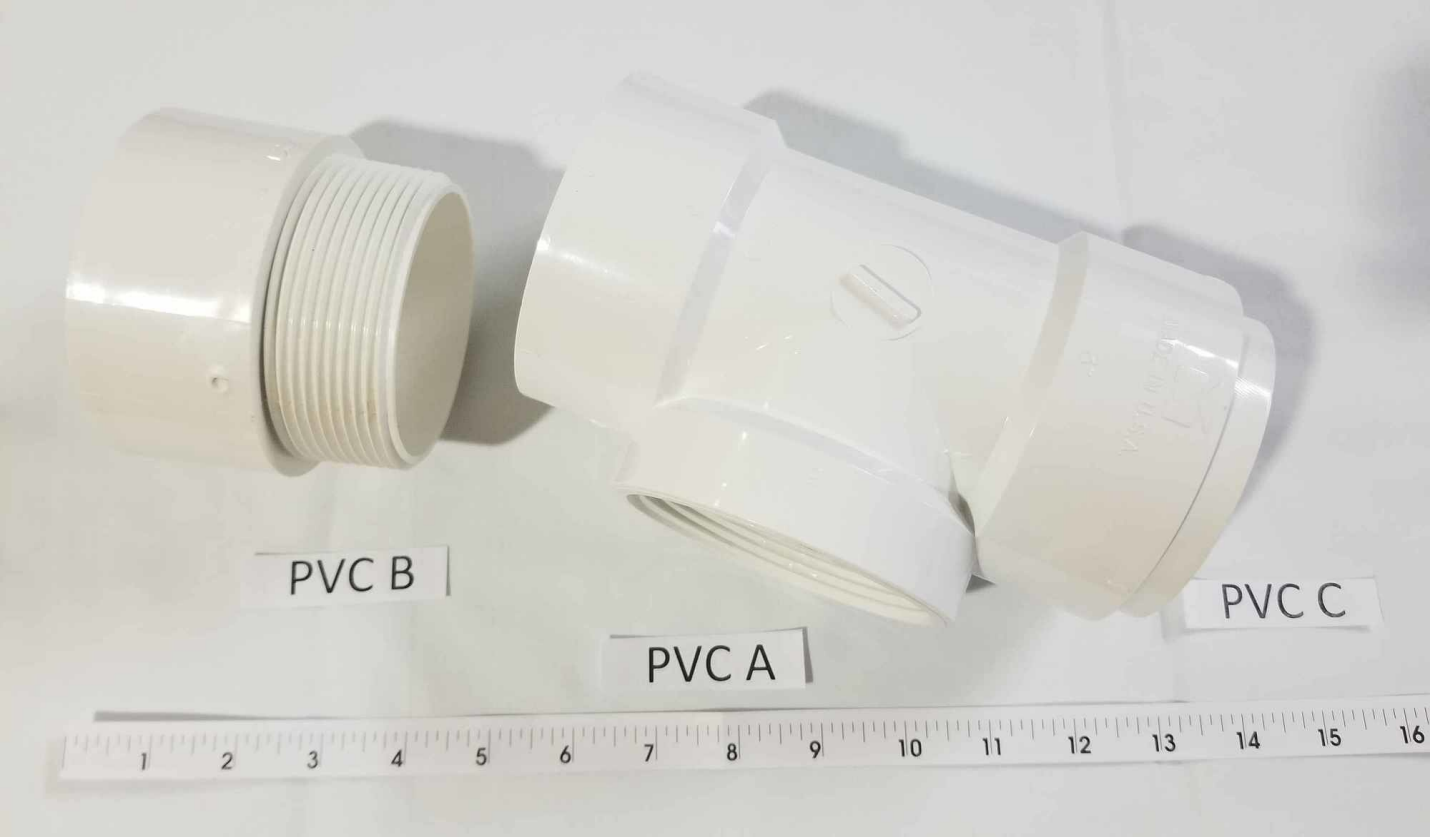
PVC pipe component of the pelvic model PVC: polyvinyl chloride A: PVC part D-2665 NSF-dwv 8415; B: 5929; C: 5907

**Figure 4:**
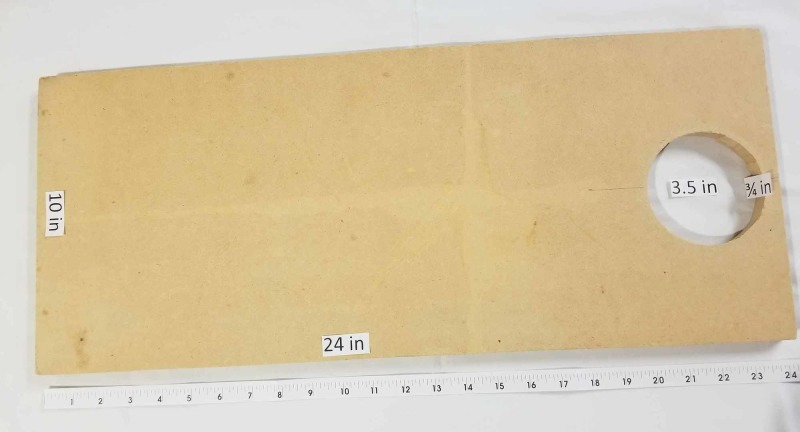
MDF board component of the pelvic model

**Figure 5:**
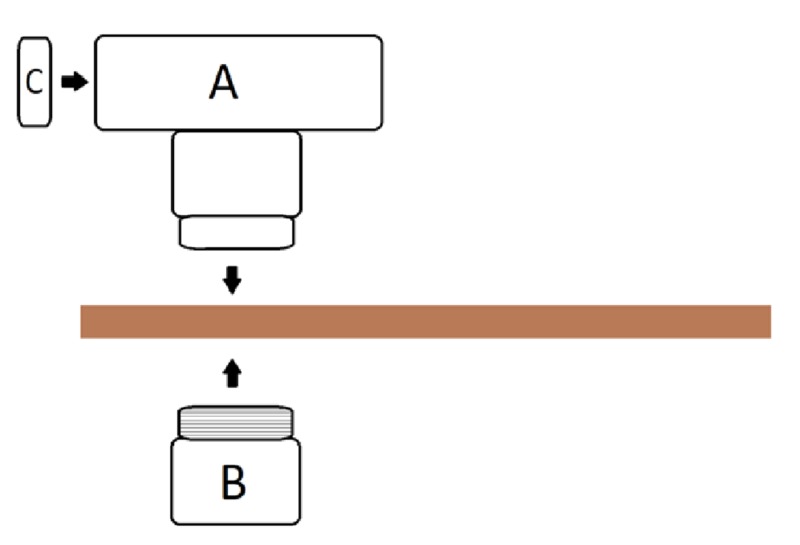
Diagram of pelvic model assembly

Multiple pelvic models were tried and the Conner model [3], where polyvinyl chloride (PVC) piping was used to simulate the pelvis and vaginal canal, was found to provide a realistic amount of resistance and working space given our uterine model. PVC piping was used to simulate the pelvis and vaginal canal. PVC parts included D-2665 NSF-dwv: 8415, 5929, and 5907 (Figure 3). A medium-density fiberboard (MDF) of 24 x 10 x 3/4 inches was used for stabilization. A 3.5-inch diameter hole was cut in the center of one end, approximately 0.5 inches from the edge (Figure 4). PVC A was then placed on top of the board through the 3.5-inch hole and connected to PVC B which was placed on the bottom of the board. PVC C was then placed within the exterior portion of the protruding pipe on PVC A (Figure 5). The reusable MDF/PVC model was built in approximately one hour and cost under USD 30.

**Figure 6:**
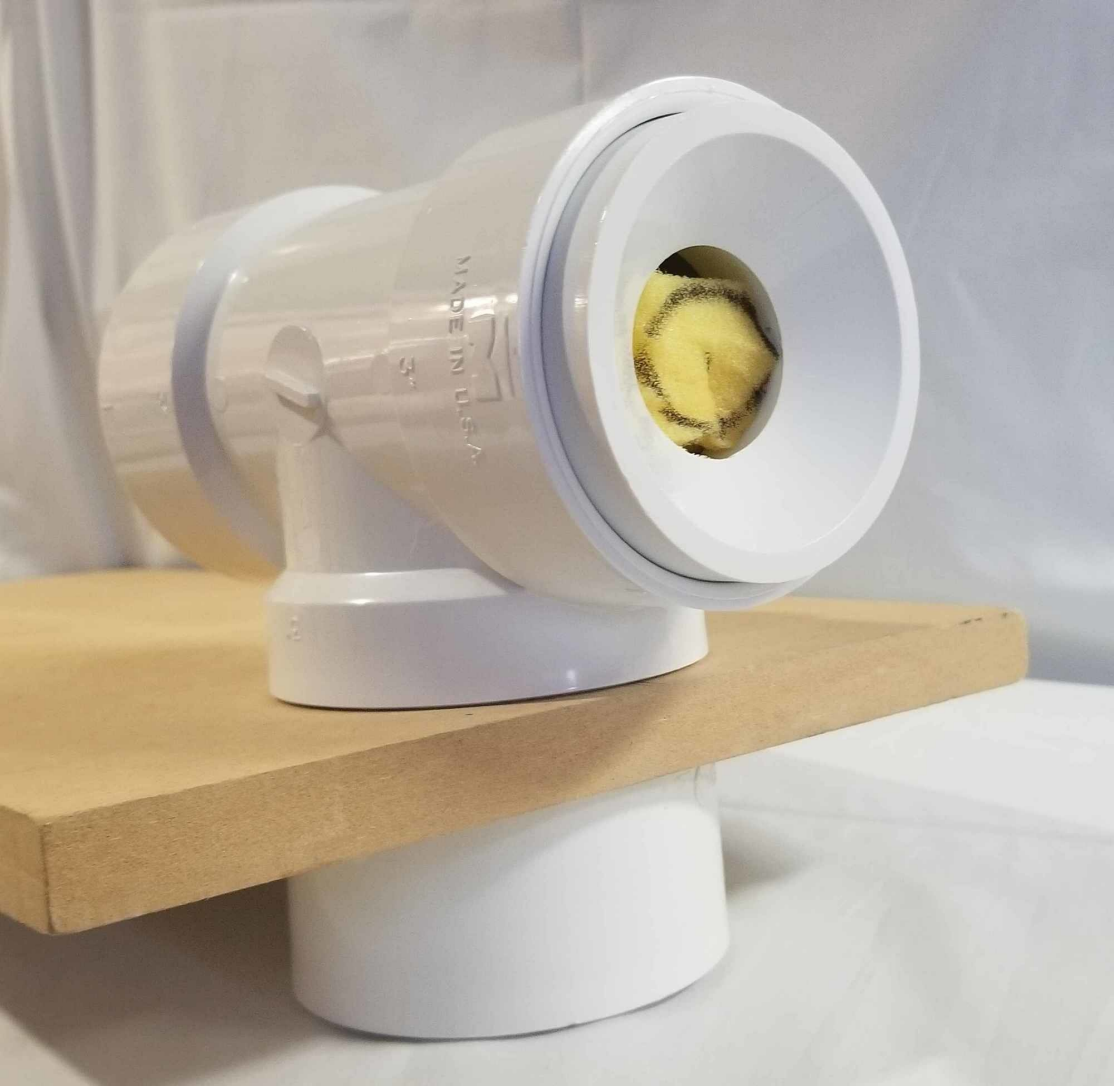
Complete assembly of the simulation

The model was then secured to a table with bungee cords or held by an observer for stabilization. The uterus model was placed into PVC A with the A for anterior marking facing upward and the cervix flush with the opening of PVC C (Figure 6). Tools supplied included two single tooth tenaculums, a scalpel, and curved mayo scissors. Instructions were to core the uterus without entering the serosa while the uterus model was inside the pelvic model.

Participants chosen to attend the simulation included attending and senior resident physicians from our department who had experience with vaginal uterine morcellation. Participants were given a survey to record their experience with the simulation. The survey specifically queried about the perceived realism of the pelvic model, the uterine model, and the overall simulation by asking participants to rate from one (very unrealistic) to five (very realistic).

## Discussion

Vaginal hysterectomy can be performed safely in most patients provided it is done by someone with proper training. Doucette et al. have reported that vaginal hysterectomy was found to be safe in patients with large uteruses, nulliparity, and patients who underwent a previous cesarean section or previous laparotomy. Transvaginal morcellation was performed in 34% of the study patients. Morcellation can be performed safely in most women including those with obesity and morbid obesity. When studied, no complications were directly attributed to transvaginal morcellation. The study group and the standard vaginal hysterectomy group had similar rates of complications [4]. As a relatively large percentage of patients required morcellation for completion of their procedure, transvaginal morcellation prevented many patients from being exposed to the morbidity of laparotomy [5].

Simulation is an important method for developing surgical confidence and competency. In a literature review of the benefits of surgical simulation, improvements in outcome were demonstrated in every study including improved technical performance [6]. As morcellation was required in one-third of the cases [4], we theorize that increasing the knowledge about and skill to perform morcellation will change mindsets when selecting patients for vaginal hysterectomy. In many studies, the size of the uterus that could be removed vaginally increased as the physicians became more experienced with coring techniques [7].

**Figure 7:**
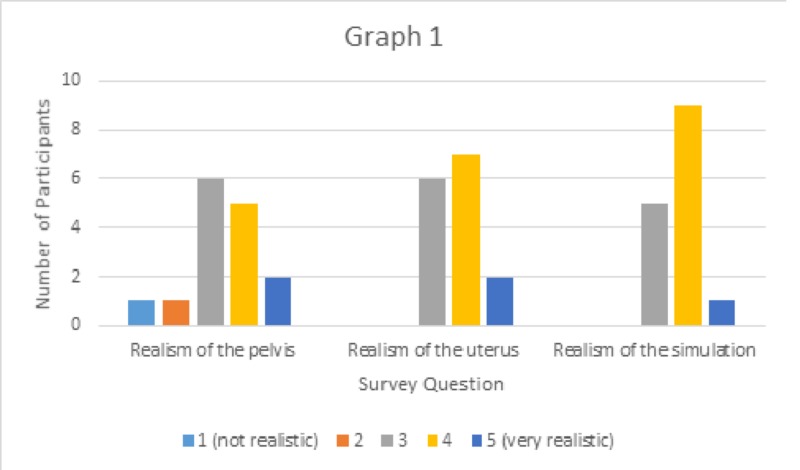
Graphical representation of the results of simulation realism survey

The simulation and subsequent survey were completed by 15 attending physicians and senior residents within our department. The realism of the pelvis was rated at an average of 3.7/5. The realism of the uterine model was rated at an average of 3.7/5, and that of the model as a whole was rated at 3.7/5 (Figure 7). Participants commented that simulation would likely turn out to be a good starting point for residents in training and it was an overall good model for vaginal morcellation. Resident physicians reported they felt more confident in their skills after the simulation. Suggestions for improvement included the lining of the pelvic portion of the model with fabric to simulate vaginal tissue that is not to be cut during morcellation. Another suggestion was to inject silicone into the foam to create intramural fibroids to be cored during morcellation.

We now plan to expand the use of our model and to look at other areas of investigation, such as whether the use of our simulation would encourage the providers to pick the vaginal route of hysterectomy more frequently. Additionally, the model could be modified to increase the breadth and depth of the simulation experience. Potential modifications for an increased variety of surgical experiences include placing a sock around the sponge model to simulate vaginal mucosa that is not to be transected, injecting silicone into the foam to simulate coring fibroids, implementing time limits, and incorporating additional morcellation procedures into the simulation curriculum.

## Conclusions

We have created a realistic model for the simulation of vaginal uterine morcellation from affordable and easily accessible materials that can be used for simulation education. On informal review, this simulation model has shown promise for teaching vaginal uterine morcellation skills. Providers have commented that the simulation is a good starting point for residents in training and provided a good overall approximation of vaginal morcellation. Resident physicians have reported that they felt more confident in their skills after participating in the simulation.
